# Supplementary material for: St. John’s Wort Suppresses Growth in Triple-Negative Breast Cancer Cell Line MDA-MB-231 by Inducing Prodeath Autophagy and Apoptosis
Source: Nutrients. 2020 Oct 17;12(10):3175. doi: 10.3390/nu12103175 (PMC7602992; doi:10.3390/nu12103175)
Supplement: Supplementary file 1 [file nutrients-12-03175-s001.pdf]

Supplemental Information

St. John's Wort suppresses the growth of triple-negative breast cancer cell line MDA-MB-231 by inducing inducing pro-death autophagy and apoptosis

Mi-Kyoung You [1], Young-Hyun Lee[2], Hwa-Jin Kim[2], Ji Hyun Kook [2], Hyeon-A Kim [2*]

[1] Department of Nutrition, University of Massachusetts Amherst, Amherst, MA,01003, USA

[2] Department of Food and Nutrition, Mokpo National University, Jeollanam-do, 58554, Republic of Korea

Supplemental Figures

Figure S1. SJWE with 3-MA for 3 days decreased proliferation in TNBC line MDA-MB-231 cells.

Figure S2. SJWE increased p-AMPK expression in TNBC line MDA-MB-231 cells.

Supplemental Tables

Table S1. List of primary antibodies

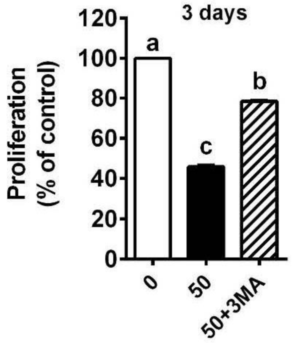

Figure S1. SJWE with 3-MA for 3 days decreased proliferation in TNBC line MDA-MB-231 cells. MDA-MB-231 cells were treated with 50 μg/mL SJWE for 3 days with or without autophagy inhibitor (3-MA, 1mM). C: DMSO (0.1% final concentration); SJWE 50: St. John's Wort ethanol extract 50 μg /mL; SJWE 50+3MA: St. John's Wort ethanol extract 50 μg /mL with 3-MA treatment. Different letters are significantly different by Duncan's multiple range test ($P < 0.05$).

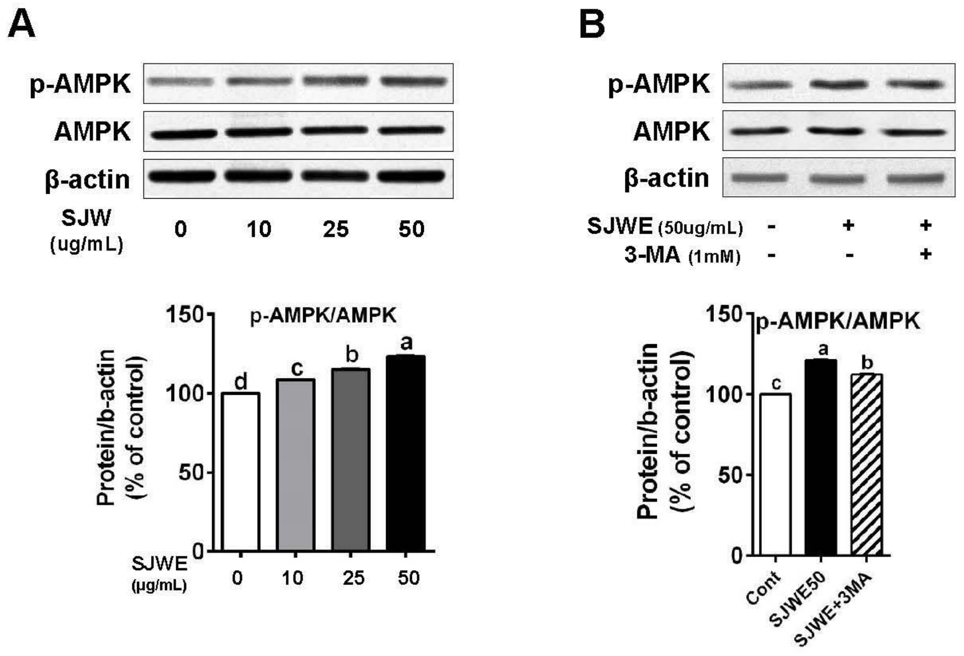

Figure S2. SJWE increased p-AMPK expression in TNBC line MDA-MB-231 cells. (**A**) MDA-MB-231 cells were treated with SJWE for 24 hours. Western bot analysis of p-AMPK and AMPK, (**B**) MDA-MB-231 cells were treated with SJWE for 24 hours with or without autophagy inhibitor (3-MA, 1mM). Western bot analysis of p-AMPK and AMPK.

Table 1. List of primary antibodies.

Antibody	Host	Dilution	Company	Catalog no.
LC3	Rabbit	1:1000	Cell Signaling	2775
p62	Rabbit	1:1000	Cell Signaling	23214
Atg3	Rabbit	1:1000	Cell Signaling	3415
Atg5	Rabbit	1:1000	Cell Signaling	12994
Atg5-12	Rabbit	1:1000	Cell Signaling	4180
Beclin 1	Rabbit	1:1000	Cell Signaling	3495
PI3K p85	Rabbit	1:1000	Cell Signaling	4292
p-PI3K p85(Tyr458)/p55(Tyr199)	Rabbit	1:1000	Cell Signaling	4228
Akt	Rabbit	1:1000	Cell Signaling	9272
p-Akt	Rabbit	1:1000	Cell Signaling	9271
Bcl-2	Rabbit	1:1000	Cell Signaling	2870
Bcl-xL	Rabbit	1:1000	Cell Signaling	2762
Bad	Rabbit	1:1000	Cell Signaling	9292
p-Bad	Rabbit	1:1000	Cell Signaling	9291
Bax	Rabbit	1:1000	Cell Signaling	2772
mTOR	Rabbit	1:1000	Cell Signaling	2983
p-mTOR	Rabbit	1:1000	Cell Signaling	5536
AMPK	Rabbit	1:1000	Cell Signaling	2532
p-AMPK	Rabbit	1:1000	Cell Signaling	23535
β-actin	Mouse	1:1000	Santa Cruz Biotechnology	sc-47778
